# Mature adipocytes in bone marrow protect myeloma cells against chemotherapy through autophagy activation

**DOI:** 10.18632/oncotarget.6020

**Published:** 2015-10-07

**Authors:** Zhiqiang Liu, Jingda Xu, Jin He, Huan Liu, Pei Lin, Xinhai Wan, Nora M. Navone, Qiang Tong, Larry W. Kwak, Robert Z. Orlowski, Jing Yang

**Affiliations:** ^1^ Department of Lymphoma and Myeloma, Division of Cancer Medicine, Center for Cancer Immunology Research, The University of Texas MD Anderson Cancer Center, Houston, Texas, USA; ^2^ Department of Pathology, The University of Texas MD Anderson Cancer Center, Houston, Texas, USA; ^3^ Department of Genitourinary Medical Oncology-Research, The University of Texas MD Anderson Cancer Center, Houston, Texas, USA; ^4^ Children's Nutrition Research Center, Baylor College of Medicine, Houston, Texas, USA

**Keywords:** multiple myeloma, adipocytes, autophagy, apoptosis, chemotherapy resistance

## Abstract

A major problem in patients with multiple myeloma is chemotherapy resistance, which develops in myeloma cells upon interaction with bone marrow stromal cells. However, few studies have determined the role of bone marrow adipocytes, a major component of stromal cells in the bone marrow, in myeloma chemotherapy resistance. We reveal that mature human adipocytes activate autophagy and upregulate the expression of autophagic proteins, thereby suppressing chemotherapy-induced caspase cleavage and apoptosis in myeloma cells. We found that adipocytes secreted known and novel adipokines, such as leptin and adipsin. The addition of these adipokines enhanced the expression of autophagic proteins and reduced apoptosis in myeloma cells. *In vivo* studies further demonstrated the importance of bone marrow-derived adipocytes in the reduced response of myeloma cells to chemotherapy. Our findings suggest that adipocytes, adipocyte-secreted adipokines, and adipocyte-activated autophagy are novel targets for combatting chemotherapy resistance and enhancing treatment efficacy in myeloma patients.

## INTRODUCTION

Multiple myeloma (MM) is characterized by a clonal expansion of malignant plasma cells [1]. It is the second most common hematological malignancy in the United States [2, 3]. With conventional treatment, the median survival duration is 3-4 years; advanced chemotherapy may extend this duration to 5-7 years or longer [4, 5]. However, most patients rapidly develop relapsed or refractory disease after undergoing high-dose chemotherapy [6, 7]. Thus, strategies to overcome chemotherapy resistance in MM patients are urgently needed.

Recent studies show that the interaction between MM cells and bone marrow stromal cells (BMSCs) induces chemotherapy resistance [8–10]. Several cell types of BMSCs, including macrophages [11, 12], plasmacytoid dendritic cells [13], osteoblasts [14, 15], osteoclasts [15–17], and immune cells [18], have been identified. These cells produce many cytokines that support MM growth and survival [19, 20]. However, the role of other types of BMSCs is unclear.

Adipocytes, which arise from mesenchymal stem cells (MSCs), are a major type of BMSCs [21–23] and the primary composes of adipose tissue. The adipocytes in white adipose tissue (WAT) contain a single large membrane-enclosed lipid droplet, surrounded by a thin rim of cytoplasm [24], and express specific proteins such as adipocyte protein 2 (aP2); they produce triglycerol [25] and adipokines [26–28], and serve as a major source of energy reserve [29]. The adipocytes in brown adipose tissue (BAT) contain a central nucleus and many small lipid droplets and mitochondria, and provide a vital source of heat to maintain body temperature. Yellow adipose tissue (YAT) has a mixed characterization and gene expression pattern of WAT and BAT. Recent studies categorize bone marrow (BM) fat as a YAT, with the abilities to initiate osteogenesis and provide energy in emergency situations [30]. In addition, an association between adipocytes and cancer was reported: in ovarian cancer, adipocytes promote rapid cancer cell proliferation; in breast cancer and leukemia, they induce tumor metastasis and inhibit apoptosis [31, 32]. Clinical studies showed an association between BM adipocytes and an increased risk of MM, although its specific biological mechanisms have yet to be elucidated [33, 34]. Adipocytes isolated from femoral BM biopsies support MM cell proliferation and migration [35]. However, no studies have determined the role of BM adipocytes in MM chemotherapy resistance.

Autophagy is a non-selective lysosomal pathway that degrades cytosolic organelles and proteins with highly regulated processes, in which the organelles and portions of cytosol are sequestered inside autophagosomes, the double-membrane vesicles, and translocated to lysosomes for fusion and content degradation. Autophagy was recently shown to play an important role in tumor development [36]. Some studies show that autophagy activation causes tumor cell death in cooperation with apoptosis [37]. Others found that autophagy reduces apoptosis by inhibiting caspase cleavage [38], suggesting a functional role in inducing cancer chemotherapy resistance. Previous studies showed that it counteracted apoptosis in MM cells, which were exposed to chemotherapy drugs *in vitro*, by regulating intracellular reactive oxygen species. However, the mechanism of autophagy activation in MM cells remains unclear. In this study, we determined the role of BM adipocytes in MM chemotherapy resistance *in vitro* and in MM mouse models. We revealed, for the first time, that adipocytes protect MM cells from chemotherapy-induced apoptosis via autophagy activation. Adipocytes upregulated the expression of autophagic proteins in MM cells *via* known adipocyte-secreted adipokines, leading to suppression of caspase cleavage and apoptosis in MM cells. Thus, we found that adipocytes in the BM, adipocyte-secreted adipokines, and adipocyte-activated autophagy are novel therapeutic targets for preventing MM chemotherapy resistance.

## RESULTS

### *In vitro* generation of BM-derived mature human adipocytes

**Figure 1:**
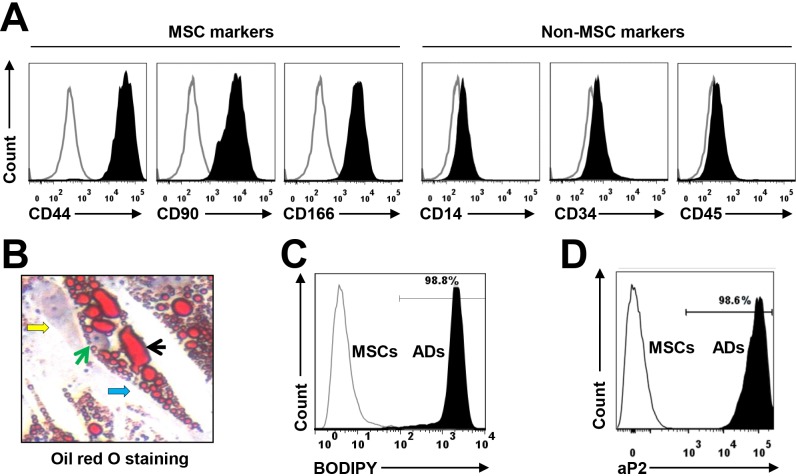
Characterization of cultured, BM-derived mature human adipocytes MSCs were derived from the BM mononuclear cells of healthy human fetal bones. **(A)** Flow cytometry analysis shows the immunophenotype of MSCs, which express the MSC-specific surface markers CD44, CD90, and CD166 but not the hematopoietic, stem cell, or endothelial protein markers CD14, CD34, and CD45. Mature adipocytes were generated from MSCs in a 2-week culture in adipocyte medium. **(B)** Shown is a mature human MSC-derived adipocyte at high magnification (x60) (indicated by a blue arrow); it contained large amounts of lipid droplets (indicated by a black arrow) in the cytoplasm, as stained with Oil red O. The green arrow points to the nucleus of the adipocyte, and a yellow arrow shows an undifferentiated MSC. Flow cytometry analysis shows the level of **(C)** BODIPY-stained and **(D)** aP2 protein (a marker of mature adipocytes)-stained adipocytes. The mature adipocytes were further sorted with the antibody against aP2 to obtain a pure adipocyte population (data not shown). Results of five independent experiments are shown.

Human MSCs were derived from BM mononuclear cells from the human fetal bones of five healthy donors or pre-adipocyte cell line PCS-210-010 (data not shown). The immunophenotype of MSCs included CD44, CD90, and CD166 but not the hematopoietic, stem cell, or endothelial markers CD14, CD34, and CD45 (Figure 1A), as determined using flow cytometry analysis. Culturing the MSCs in adipocyte medium for 2 weeks induced mature adipocyte formation, whereas culturing them in MSC medium did not. The generated mature adipocytes contained large amounts of lipid droplets in the cytoplasm, as detected with Oil Red O staining (Figure 1B); produced high levels of soluble triglycerol, as determined by a BODIPY assay (Figure 1C); and expressed the specific adipocyte differentiation-associated protein aP2, as determined by flow cytometry (Figure 1D); the undifferentiated cells did not. To avoid trace undifferentiated cell effects, the mature adipocytes were further purified for the studies below.

### BM-derived adipocytes protect MM cells from chemotherapy-induced apoptosis

**Figure 2:**
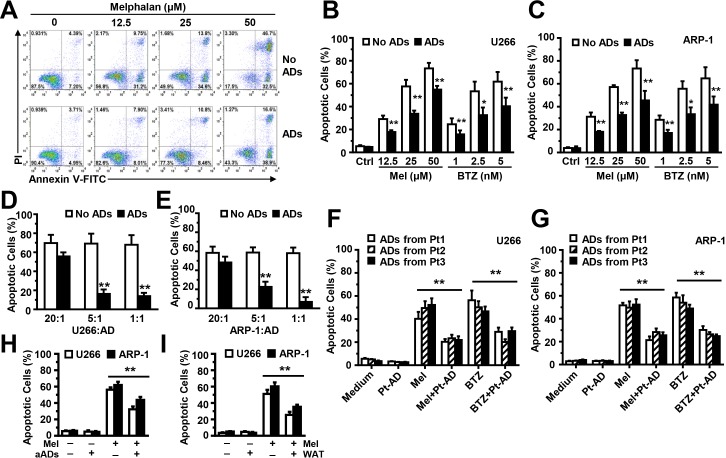
Co-culture with adipocytes protects MM cells from melphalan-induced apoptosis *in vitro* **(A)** The representative histograms show the percentages of apoptotic ARP-1 cells in 24-hour co-cultures, without or with adipocytes at a ratio of 5:1 in medium with melphalan (0, 12.5, 25, or 50 μM). Summarized data are shown in **(B)** for U266 cells and **(C)** for ARP-1 cells treated with melphalan or bortezomib. Also shown are the percentages of apoptotic U266 **(D)** and ARP-1 **(E)** cells, cultured alone (no adipocytes) or with mature adipocytes at a ratio of 20:1, 5:1, or 1:1 for 24 hours. Apoptotic cells were detected using an Annexin V-binding assay. Mature adipocytes were generated from the MSCs derived from the BM mononuclear cells of three healthy human fetal bones **(A-E)** Similar experiments were done co-culture of U266 cells **(F)** or ARP-1 cells **(G)** with mature adipocytes isolated from bone marrow aspirate of three MM patients at a ratio of 5:1 for 24 hours, as well as with adipocytes isolated from adult BM aspirates (aADs; **H**) or from WAT cells (WAT; **I**). Results of five independent experiments are shown. **P < 0.01.

To determine whether BM-derived adipocytes have a functional role in chemotherapy-induced MM cell apoptosis, we seeded ARP-1 cells, without or with adipocytes, at a ratio of 5:1 in medium; different doses of melphalan were added for 24 hours. Compared with the ARP-1 cell culture alone, cells co-cultured with adipocytes had a much lower percentage of apoptosis (Figure 2A). Similarly, co-culture with adipocytes inhibited apoptosis in U266 (Figure 2B) or ARP-1 (Figure 2C) cells induced by melphalan or bortezomib. Increasing adipocyte numbers in the co-cultures had an increased ability in a dose-dependent manner to reduce apoptosis of MM cells U266 (Figure 2D) and ARP-1 (Figure 2E) induced by melphalan. Co-culture with MM patients' adipocytes (Figure 2F and 2G), adult adipocytes (Figure 2H), or WAT cells (Figure 2I) offers the protection as well.

**Figure 3:**
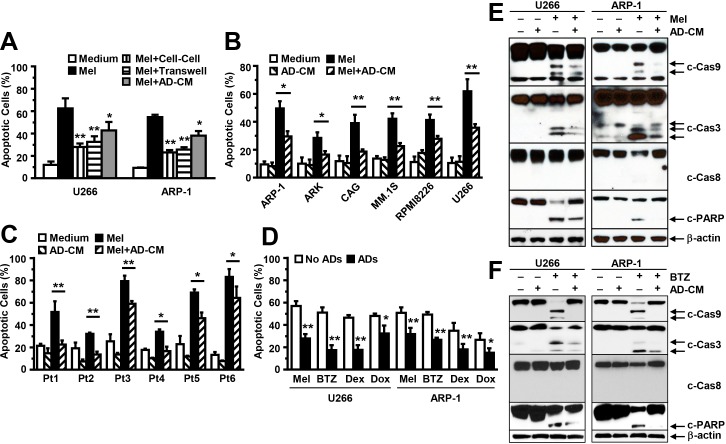
Addition of adipocyte-conditioned media (CM) inhibits MM cell apoptosis **(A)** Annexin V-binding assay shows the percentages of apoptotic U266 and ARP-1 cells co-cultured with adipocytes together (cell-cell), co-cultured with adipocytes but separated by transwell inserts (Transwell), or cultured in medium containing adipocyte-CM in the presence of the drug melphalan (Mel; 25 μM). annexin V-binding assay shows the percentages of apoptotic cells in **(B)** the MM cell lines ARP-1, ARK, CAG, MM.1S, RPMI8266, and U266; and **(C)** primary MM cells isolated from the BM aspirates of six MM patients (Pt1 to Pt6) cultured in medium alone, adipocyte-CM, or melphalan (Mel; 25 μM), or a combination (adipocyte-CM+Mel); and **(D)** the percentages of apoptotic U266 and ARP-1 cells cultured with or without (medium) adipocyte-CM and containing melphalan (Mel), bortezomib (BTZ), dexamethasone (DEX), or doxorubicin (Dox). Cells were cultured for 24 hours. Purified mature adipocytes were generated from the MSCs derived from five healthy human fetal bones. Results of five independent experiments are shown. *P ≤ 0.05; **P ≤ 0.01. Western blot analysis shows the reduced levels of cleaved caspase-9 (c-Cas9), caspase-3 (c-Cas3), and PARP and unchanged levels of caspase-8 and β-actin in U266 and ARP-1 cells that had been cultured in medium containing adipocyte-CM or **(E)** melphalan (Mel) or **(F)** bortezomib (BTZ), alone or in combination. Results of three independent experiments are shown.

Previous studies showed that adipocytes that are in direct contact with tumor cells can transfer energy-containing lipids into the cells to promote their proliferation [32]. To determine whether adipocytes inhibit MM cell apoptosis by coming into direct contact with MM cells, we co-cultured MM cells and adipocytes and separated them, using transwell inserts [39], in a medium containing melphalan. The MM cells were still protected from the apoptotic effects of melphalan (Figure 3A). Previous studies also showed that adipocytes can take up drugs, thereby decreasing the drugs' effects on tumor cells [40]. To avoid the possibility that adipocytes inhibit MM cell apoptosis by absorbing drugs, we cultured purified adipocytes for 24 hours, collected the adipocyte-conditioned media, and added the adipocyte-conditioned media plus melphalan to the MM cell culture. Because the culture had no adipocytes, no drug absorption occurred. Our results showed that MM cells were still protected against melphalan treatment (Figure 3A). We cultured other MM cell lines (Figure 3B) and primary MM cells isolated from the BM aspirates of six MM patients (Figure 3C) in medium with adipocyte-conditioned media. Culturing MM cells with adipocyte-conditioned media protected them from the treatments of melphalan, bortezomib, dexamethasone, and doxorubicin (Figure 3D). Western blot analysis further showed that melphalan treatment enhanced the levels of cleaved caspase-9 and -3, but not -8, and PARP in U266 and ARP-1 cells, while the addition of adipocyte-conditioned media significantly reduced melphalan (Figure 3E) or bortezomib (Figure 3F)-induced caspase-9, -3, and PARP cleavage.

### Adipocytes activate autophagy and inhibit apoptosis in MM cells

**Figure 4:**
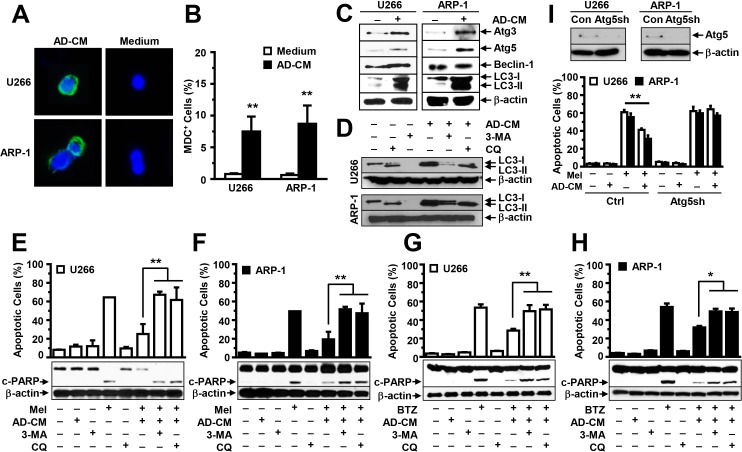
Addition of adipocyte- conditioned media (CM) activates autophagy in MM cells **(A)** Representative images of MDC-stained ARP-1 and U266 cells in medium, with or without adipocyte-CM, at high magnification (×60). **(B)** MDC-positive cells were counted under the microscope, and the percentages of MDC-positive cells were plotted. **(C)** Western blot analysis shows the enhanced expression of autophagy proteins Atg3, Atg5, and LC3-I/II, but not Beclin-1, in U266 and ARP-1 cells after 24 hours of culture in medium, with or without adipocyte-CM (AD-CM). Moreover, **(D)** Western blot analysis shows the reduced expression of autophagy proteins LC3-I/II in U266 and ARP-1 cells cultured in medium with or without adipocyte-CM and treated with or without autophagy inhibitors 3-methyladenine (3-MA; 1 mM) or chloroquine (CQ; 50 μM). In addition, Western blot analysis (lower panels) shows the levels of cleaved (c-) PARP, and β-actin, and an annexin V-binding assay (upper panels) shows the percentages of apoptosis in U266 **(E**, **G)** and ARP-1 cells **(F**, **H)** in 24-hour cultures treated with **(E**, **F)** melphalan (Mel) or **(G**, **H)** bortezomib (BTZ), in the presence or absence of adipocyte-CM. Autophagic protein Atg5 was knocked down in ARP-1 and U266 cells by using lentivirus-carried Atg5 shRNAs. **(I)** Western blot analysis (upper panels) shows the reduced Atg5 expression and annexin V-binding assay (lower panels) shows the enhanced apoptosis in Atg5 shRNA-MM cells as compared with non-target control MM cells. Addition of AD-CM to the cultures at a ratio of 1:5 is shown. β-actin levels served as an internal loading control. Results of three independent experiments are shown. **P ≤ 0.01.

Previous studies showed that autophagy activation inhibits caspase cleavage, inducing chemotherapy resistance in many tumors. Thus, we determined whether adipocytes activate autophagy in MM cells. U266 and ARP-1 cells were cultured in medium, with or without adipocyte-conditioned media, and the MDC assay was used to detect autophagy. We observed a significant increase in autophagy in adipocyte-conditioned media-treated MM cells but little autophagy in non-treated cells (Figure 4A and 4B). Western blot analysis further showed that the addition of the adipocyte-conditioned media significantly upregulated the expression of the autophagy proteins Atg3, Atg5, and LC3-I/II but not Beclin-1 (Figure 4C). The addition of conditioned media of adipocytes generated from the pre-adipocyte line (Figure 4A-C) or primary MSCs of five healthy human fetal bones (data not shown) had a similar effect. To determine whether adipocyte-induced autophagy mediates adipocytes' protection of MM cells from apoptosis, we added specific inhibitors against autophagy 3-methyladenine or chloroquine to the MM cell culture containing adipocyte-conditioned media in presence or absence of melphalan or BTZ. Treatment with both inhibitors reduced autophagy (Figure 4D), and enhanced PARP cleavage and apoptosis in melphalan (Figure 4E and 4F) or BTZ (Figure 4G and 4H) treated U266 (Figure 4E and 4G) and ARP-1 cells (Figure 4F and 4H), even in adipocyte-conditioned media. Atg5 is a key protein which is required for autophagy activation. Thus, we knocked down Atg5 expression in ARP-1 and U266 cells using Atg5 shRNAs (Figure 4I; upper panels). Cultures of non-target shRNA-, but not Atg5 shRNA-MM cells, with adipocyte-conditioned media inhibited melphalan-induced apoptosis (Figure 4I; lower panels), indicating that autophagy mediates adipocyte protection.

### Adipocytes produce soluble adipokines that increase the expression of autophagy proteins

**Figure 5:**
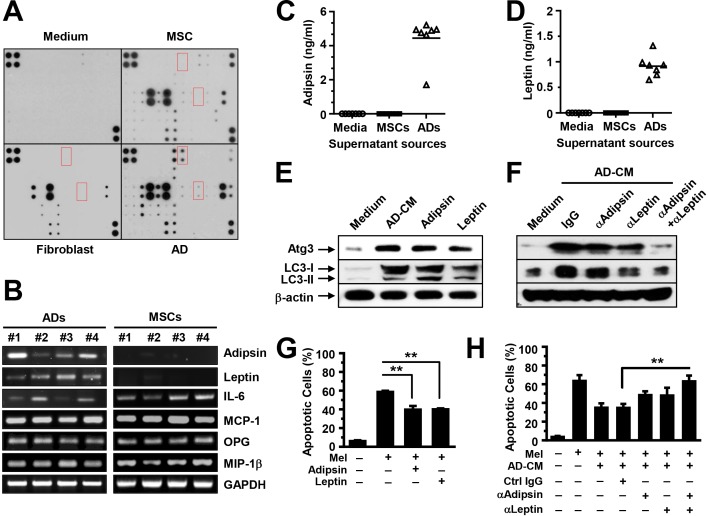
Adipocytes produce adipokines that activate autophagy **(A)** Representative images of arrays show the profile of adipokine expression in the supernatants of cultured adipocytes. Medium and the supernatants from cultured MSCs or fibroblasts served as controls. We performed quantitative real-time PCR to determine the adipocyte-expressed **(B)** and ELISA to determine the adipocyte-secreted adipsin **(C)** and leptin **(D)**, and the expression and secretion in MSCs served as controls. **(E)** Western blot analysis shows the increased expression of the autophagy proteins Atg3 and LC3-I/II and unchanged β-actin in U266 cells in 24-hour culture in medium with adipocyte-conditioned media (AD-CM) or the adipokines adipsin (100 ng/ml) or leptin (25 ng/ml). Neutralizing antibodies against adipsin (2 μg/ml) or leptin (2 μg/ml), alone or in combination, were added to U266 cells in medium with adipocyte-CM. Addition of isotype IgG served as control. **(F)** Western blot analysis shows the reduced expression of the autophagy proteins Atg3 and LC3-I/II in U266 cells treated with the neutralizing antibodies. Antibody combination had a synergistic reduction in the autophagy protein expression as compared with treatment alone. **(G)** Annexin V-binding assay showed the percentage of apoptotic U266 cells in cultures with melphalan, and with or without adipsin (100 ng/ml) or leptin (25 ng/ml). **(H)** Annexin V-binding assay shows the percentage of apoptotic U266 cells in cultures with adipocyte-CM, with or without neutralizing antibodies in the presence or absence of melphalan. Representative results from three experiments are shown. **P ≤ 0.01.

We elucidated the mechanism by which adipocytes activate autophagy. We first analyzed adipocyte-secreted soluble factors using an array containing specific antibodies against 62 adipokines that are known to be produced by adipocytes. Culture medium and the supernatants from MSCs or fibroblasts served as controls. We observed the adipokines highly presented in the supernatant of the adipocytes (Figure 5A). Furthermore, we collected the mRNAs and conditioned media of cultured MSCs and the purified mature adipocytes generated from MSCs, and determined adipokine expression and secretion using quantitative RT-PCR (Figure 5B) and ELISA (Figure 5C-5D). Our results showed that adipokines such as leptin and adipsin were produced mainly by adipocytes, whereas other adipokines, such as interleukin (IL)-6, monocyte chemoattractant protein (MCP)-1, osteoprotegerin (OPG), and macrophage inflammatory protein (MIP)-1β, were produced by both adipocytes and MSCs. To determine whether adipokines activate autophagy, we added recombinant adipsin and leptin, which are adipocyte derived, to MM cell cultures. The addition of IL-6, which is both adipocyte and MSC derived, served as a control. Western blot analysis results showed that the addition enhanced the expression of Atg3 and LC3-I/II in MM cells (Figure 5E). Moreover, anti-adipsin or anti-leptin antibodies, alone or in combination, or control IgG, were added to U266 cells in medium with adipocyte-conditioned media. Our results showed the reduced Atg3 and LC3-I/II in U266 cells treated with either anti-leptin or anti-adipsin antibody. Addition of both antibodies had a synergistic effect as compared with the antibody alone (Figure 5F). Culture with leptin or adipsin protected MM cells against melphalan-induced apoptosis (Figure 5G), and addition of anti-leptin and/or anti-adipsin antibodies reduced adipocyte-induced protection (Figure 5H).

### Adipocytes activate MM cell autophagy *via* Jak/Stat3 signaling

**Figure 6:**
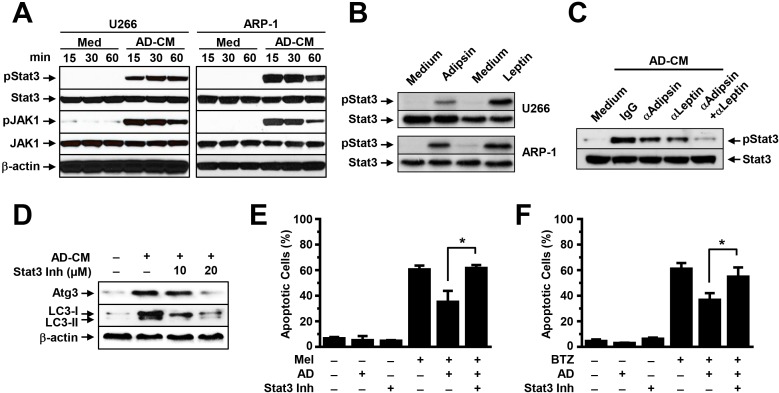
Adipocytes activate autophagy *via* the Stat3 signaling pathway Western blot analysis shows **(A)** the enhanced phosphorylated (p) and unchanged non-phosphorylated levels of Stat3 and Jak1 in U266 and ARP-1 cells cultured in medium with or without adipocyte-conditioned media (CM) for 15, 30, and 60 minutes (min), **(B)** the levels of pStat3 in U266 and ARP-1 cells 24 hours after addition of leptin (25 ng/ml) or adipsin (100 ng/ml), and **(C)** the levels of pStat3 in 24-hour culture of ARP-1 cells with adipocyte-CM and control IgG or the neutralizing antibodies again adipsin (2 μg/ml) or leptin (2 μg/ml), alone or both. Shown are **(D)** Western blot analysis results for levels of the autophagy proteins Atg3 and LC3-I/II and unchanged β-actin and annexin V-binding assay results for the percentages of apoptotic ARP-1 cells treated with melphalan **(E)** or bortezomib **(F)** in 24-hour culture in medium, without or with adipocyte-CM, Stat3 inhibitor, or both (Stat3 Inh; 10 μM and 20 μM). **P ≤ 0.01. Representative results from three experiments are shown.

We determined which signaling pathway mediates adipokine-activated autophagy. We focused on Jak/Stat3 because many adipokines, such as leptin, activate signaling in MM cells and this signaling has been shown to support MM cell proliferation and survival. To determine the effects of adipocytes on Jak/Stat3 activation, we added adipocyte-conditioned media, leptin, or adipsin to cultures of ARP-1 and U266 cells. No addition of adipocyte-conditioned media (Medium) served as a control. Western blot analysis results showed that the addition of adipocyte-conditioned media upregulated the levels of pStat3 and pJak1 in a time-dependent manner (Figure 6A). Addition of leptin or adipsin could also upregulated pStat3 levels (Figure 6B), while blocking leptin or adipsin by neutralizing antibodies reduced pStat3 levels (Figure 6C). The levels of non-phosphorylated kinases and β-actin protein were unchanged. Furthermore, the addition of a specific inhibitor against Stat3 to the MM cell culture containing adipocyte-conditioned media reduced the expression of the autophagy proteins Atg3 and LC3-I/II (Figure 6D) and enhanced melphalan (Figure 6E) or bortezomib (Figure 6F)-induced apoptosis in MM cells.

### Adipocytes protect MM cells from chemotherapy and reduce MM cell apoptosis *in vivo*

**Figure 7:**
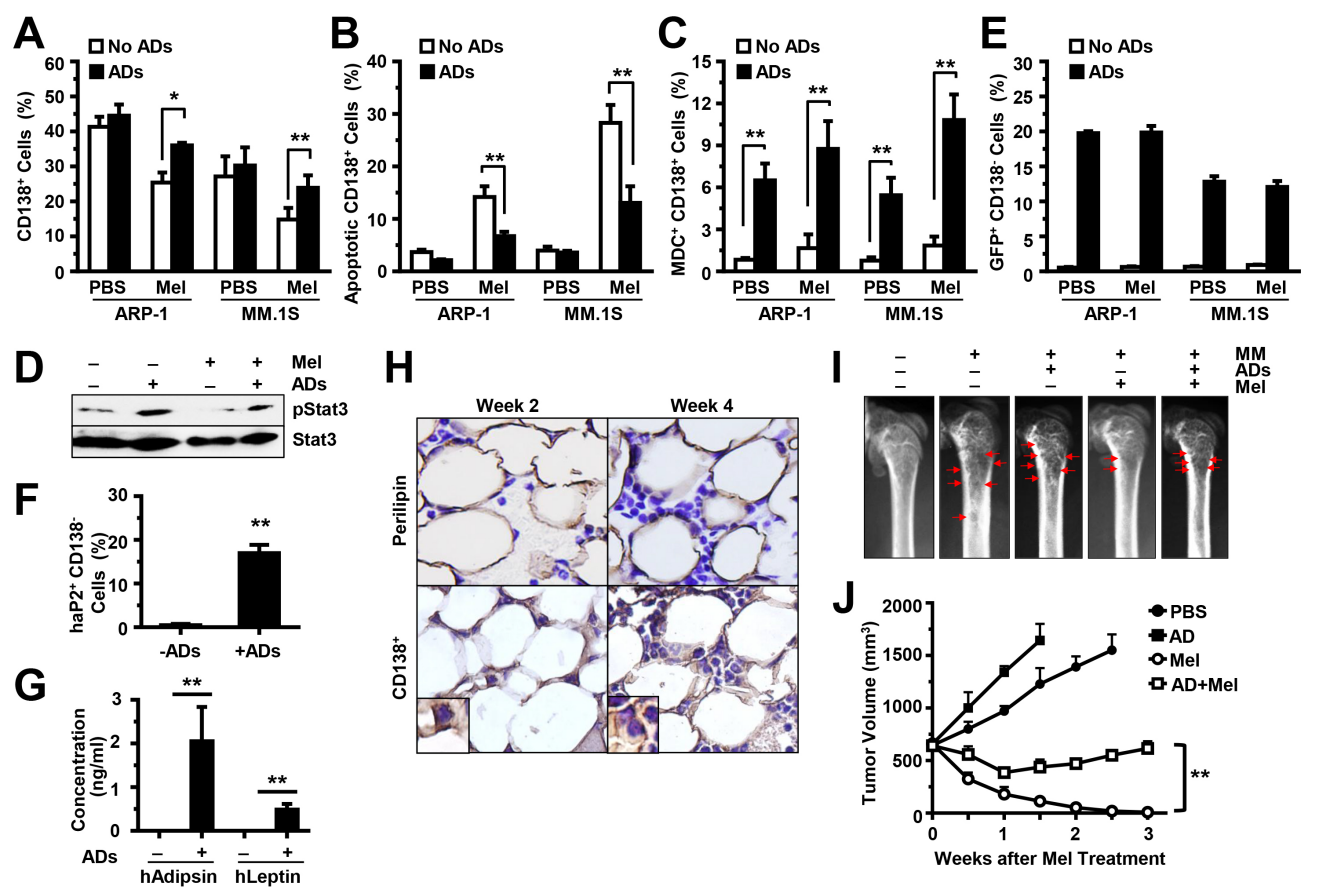
Adipocytes inhibit MM cell apoptosis and induce MM drug resistance *in vivo* SCID mouse femurs were injected with MM cells ARP-1 or MM.1S or co-injected with MM cells and GFP-labeled mature human adipocytes, and the mice were treated with or without melphalan (Mel). After treatment, BM cells from SCID mouse femurs were flushed out and labeled with anti-human CD138 antibody. The percentages of human CD138^+^ cells are shown in **(A)**. The cells were further sorted by anti-human CD138-coated magnetic beads. An aliquot of CD138^+^ cells was examined with the annexin V-binding assay for apoptosis, and the percentages of apoptotic cells are shown in (**B).** An aliquot of CD138^+^ cells was stained with the MDC assay for autophagy, and the percentages of MDC^+^ cells are shown in **(C).** An aliquot of CD138^+^ cells isolated from the BM aspirates of ARP-1 tumor-bearing mice was examined with the Western blot analysis and the levels of phosphorylated (p) Stat3 are shown in **(D)**. In addition, the aliquot of CD138^−^ cells was labeled with anti-GFP antibody to detect the injected GFP-carried adipocytes **(E)** or with anti-human aP2 antibodies to detect injected adipocytes **(F)**, and counted by flow cytometry. **(G)** Concentration of human leptin or human adipsin in the serum of mouse injected with or without human adipocytes as measured by ELISA. (**H)** Representative images of immunohistochemical staining show the expression of perilipin (an adipocyte marker) and CD138 (a MM marker) in the BM of SCID mice bearing ARP-1 cells with mature adipocytes at week 2 or week 4 post cell injection. **(I)** Representative images of radiography show the osteolytic bone lesions in the femurs of the ARP-1 tumor-bearing mice four weeks post cell injection. The image from the mice without MM cells served as control. Red arrows indicate osteolytic lesions. In addition, MM ARP-1 cells with or without mature adipocytes (ADs) were subcutaneously injected into SCID mice. 4 weeks after the cell injection, mice were intraperitoneally injected with 50 μg/mouse of melphalan (Mel) or PBS (control), twice a week for 3 weeks. **(J)** Shown are tumor volume changes in the mice after the treatment. The results represent average values from three independent experiments of five mice per group. **P < 0.01.

To determine the *in vivo* effects of adipocytes on chemotherapy resistance, we injected ARP-1 or MM.1S cells, alone or with GFP-labeled, purified mature adipocytes (generated from pre-adipocyte PCS-210-010), directly into the femurs of SCID mice. Two weeks later, the mice were intraperitoneally injected with melphalan, twice a week, for a total of four injections. Mouse BM cells (including the MM cells, the injected GFP-labeled adipocytes, and the unlabeled murine BMSCs) were flushed out. Our results showed that after melphalan treatment, the mouse femurs co-injected with MM cells and adipocytes had larger numbers of CD138^+^ cells; this reflects the MM tumor burdens in these mice, compared with those in mice injected with MM cells alone (Figure 7A). After anti-CD138 antibody sorting, the CD138^+^ cells were subjected to an annexin V-binding assay to assess apoptosis, to a MDC assay to assess autophagy, and to a Western blot analysis to assess Stat3 phosphorylation in MM cells. We found a lower percentage of apoptosis (Figure 7B), higher percentage of autophagy (Figure 7C), and upregulated levels of pStat3 (Figure 7D) in CD138^+^ MM cells from mice injected with MM cells and adipocytes than from those injected with MM cells only.

The CD138^−^ cells were labeled with anti-GFP antibody, and the GFP^+^ cells were counted by flow cytometry to assess the injected GFP-labeled human adipocytes. We only observed GFP^+^ cells in mice injected with human adipocytes. There was no difference in GFP+ cell numbers between melphalan-treated mice and PBS-treated mice (Figure 7E). Moreover, flow cytometry analysis showed the expression of human aP2 in the GFP+ cells (Figure 7F), and ELISA showed the secretion of human leptin and adipsin in the serum of mice injected with human adipocytes (Figure 7G), indicating that the injected adipocytes are functional in the BM. To validate the status of adiposity and tumor burden in BM, the mouse femurs were collected at week 2 and week 4 (after cell injection). Perilipin is a lipid droplet coat protein and a marker for adipocytes. The femurs were immunohistochemically stained with anti–perilipin antibody for adipocytes, or with anti-CD138 antibody for MM cells. Perilipin^+^ cells were present in the BM of mice injected with human adipocytes (Figure 7H). There was no change in perilipin+ cell numbers between week 2 and week 4, with increased numbers of CD138^+^ cells at week 4 (Figure 7H). Furthermore, radiography was used to examine bone lesion in mouse femurs at week 4. In untreated mice, there was little difference of bone lesion in MM-bearing mice with and without adipocytes. With melphalan treatment, more bone lesions were observed in MM-bearing mice with adipocytes than the one without adipocytes (Figure 7I). In the xenograft subcutaneous MM mouse models, with melphalan treatment, more tumor volumes were observed in MM-bearing mice with adipocytes than the one without adipocytes (Figure 7J). Taken together, the mouse studies reveal that adipocytes support MM growth and induce chemotherapy resistance *in vivo*.

## DISCUSSION

In this study, we elucidated a novel mechanism of MM chemotherapy resistance. We revealed that adipocytes, a BMSC that heavily infiltrates MM patients' BM, are important in the induction of chemotherapy resistance.

Recent studies show that direct tumor cell-adipocyte contact leads to lipids transferring from adipocytes to tumor cells via aP2, where they provide energy to tumor cells. In this study, the separation of MM cells and adipocytes using the transwell inserts in the co-culture did not significantly reduce adipocytes' protective effects against drug-induced apoptosis. In addition, adipocytes have been shown to take up drugs, decreasing the drugs' efficacy at killing tumor cells [40]. We found that the addition of adipocyte-conditioned media had a protective effect, indicating that adipocytes inhibited MM cell apoptosis at least partly *via* soluble factors. In the conditioned media of adipocytes generated from BM-derived primary MSCs or a pre-adipocyte cell line, we observed soluble cytokines and adipokines: some of these are mainly produced by adipocytes, such as leptin and adipsin; others are produced by either adipocytes or other BMSCs. A previous study showed that MM cells expressed a leptin receptor, and BM adipocytes secreted leptin. Addition of leptin resulted in a minor increase of MM cell proliferation *in vitro* [35]. Our study discovered a novel impact of adipocyte-secreted leptin in MM chemotherapy resistance. However, the role of other adipokines, such as adipsin, in MM pathogenesis is unknown. Adipsin is involved in the alternative complement pathway of the complement system. It is a serine protease that stimulates glucose transport for triglyceride accumulation in fat cells and inhibits lipolysis; its levels are elevated in the obese people. In our study, the adipocyte-secreted adipsin inhibited chemotherapy-induced MM cell apoptosis, suggesting a novel mechanism by which adipocytes induce chemotherapy resistance *via* secreted known and novel soluble adipokines.

Autophagy plays a dual role in tumor development: while autophagy can cause tumor cell death;[36] it can also support tumor cell survival and induce chemotherapy resistance.[41, 42] Recent studies show that activation of autophagy contributes to carfilzomib resistance in MM through AMPK-dependent autophagosome formation.[43] In addition, KLF4-SQSTM1/p62-mediated autophagy resists against bortezomib treatment [44], and inhibition of autophagy overcomes the resistance.[45] Our study revealed that adipocytes induced autophagy activation *via* derived adipokines; adipocyte-activated autophagy is a novel mechanism of MM chemotherapy resistance. We demonstrated that adipsin and leptin upregulate autophagic protein expression. Autophagy inhibition counteracted adipocytes' inhibition of caspase cleavage and apoptosis in MM cells. Recent studies show regulation of autophagy by several signaling pathways. Interaction of Akt/mTOR or Bcl-2 with Beclin-1 suppresses the accumulation of autophagy proteins, but JNK and NF-κB bind to the promoters of Beclin-1 and LC3-I/II and transcriptionally regulate gene expression. We revealed an additional pathway by which Jak/Stat3 signaling, activated by adipocyte-secreted adipokines, upregulated the expression of LC3-I/II, Atg3, and Atg5 but not Beclin-1. Stat3 inhibitors disrupted adipocyte-induced autophagy activation. However, the mechanism by which Stat3 regulates autophagy protein expression needs further investigation. Our results indicate that BM adipocytes, adipokines, and adipocyte-activated autophagy are novel targets for combatting chemotherapy resistance. We can also broaden our research with additional chemotherapy drugs, such as thalidomide and lenalidomide, to enhance the efficacy of current treatment and improve the overall outcomes in MM patients.

## MATERIALS AND METHODS

### Reagents and antibodies

Unless otherwise stated, BODIPY 493/503 from Life Technologies, chemicals from Sigma-Aldrich, antibodies for flow cytometry analysis from BD Biosciences, and antibodies for Western blot analysis from Cell Signaling Technology, were purchased.

### MM Cell lines and primary MM cells

ARP-1 and ARK cells were kindly provided by Arkansas Cancer Research Center, AR. Others were purchased from American Type Culture Collection (ATCC). Primary MM cells were isolated from BM aspirates of MM patients using anti-CD138 antibody-coated magnetic beads (Miltenyi Biotec, Inc.). Cells were maintained in RPMI1640 medium with 10% fetal bovine serum (FBS). This study was approved by the institutional review board of The University of Texas MD Anderson Cancer Center (Houston, Texas).

### *In vitro* generation, isolation, and characterization of human MSCs and mature adipocytes

BM cells were flushed out from human fetal femoral bones (Advanced Bioscience Resources), and cultured in DMEM to generate MSCs [46]. Immunophenotyping of MSCs was analyzed by flow cytometry. To generate mature adipocytes, MSCs were cultured in adipocyte medium (Lonza) for 2 weeks. Adipocytes were also generated from the adipocyte precursor (pre-adipocyte) cell line PCS-210-010 (ATCC), or pre-WAT cells isolated from subcutaneous fat (LONZA), or isolated from the BM aspirates of healthy adults or MM patients, and further purified by 10 min centrifugation at 186 g [47]. Mature adipocytes were fixed with 4.0% paraformaldehyde, stained with Oil red O for 1 hour, and observed under light microscopy [48].

### Co-cultures of MM cells and mature adipocytes

MM cells (1×10^5^/ml) were seeded in culture wells that had been coated with mature adipocytes (2×10^4^/ml); alternatively, adipocytes were seeded in the culture wells and MM cells were seeded in the transwell inserts in RPMI 1640 medium. Adipocyte-conditioned media was added to the cultures of MM cells. In some experiments, 25 μM melphalan, 10 μM dexamethasone, 5 nM bortezomib, or 1 μM doxorubicin was added for 24 hours. DMSO served as the control.

### Lentiviral infection of MM cells with short-hairpin RNA (shRNA)

MM cells were infected by lentivirus containing non-target shRNA or human Atg5 shRNA (Sigma-Aldrich) according to the manufacturer's protocol. Stable cell lines were selected with 0.7 μg/ml of puromycin (Sigma-Aldrich) for 4 weeks.

### Identification of adipokines in culture medium by an array and ELISA

The supernatants (as conditioned media) were collected from cultures of MSCs, fibroblasts (ATCC), or adipocytes in serum-free DMEM medium for 24 hours. The array (Raybio Human Obesity Array C1 kit; RayBiotech, GA) was incubated with conditioned media and analyzed according to the manufacturer's instructions. The levels of adipokines in conditioned media were measured by enzyme-linked immunosorbent assay (ELISA) kit (R&D Systems, Inc.).

### Apoptosis assay

An annexin V-binding assay was used to detect cell apoptosis, according to the manufacturer's instructions, and analyzed by a BD LSRFortessa flow cytometer.

### Monodansyl cadaverine (MDC) staining

Cells were stained with 100 μM MDC at 37°C for 15 minutes, and analyzed under a fluorescence microscope (Olympus BX60) [39]. The percentage of MDC+ cells was determined by a score of 500 cells per sample.

### Quantitative real-time polymerase chain reaction

Total RNA was isolated using the RNeasy kit (Qiagen) and reverse transcribed. The cDNAs were amplified with the StepOnePlus real-time PCR system (Life Technologies), SYBR Green, and the following primers:

*adipsin*: GACACCATCGACCACGACC, GCCACGTCGCAGAGAGTTC;

*leptin*: TGCCTTCCAGAAACGTGATCC, CTCTGTGGAGTAGCCTGAAGC;

*Interleukin* (IL)-6: ACTCACCTCTTCAGAACGAATTG, CCATCTTTGGAAGGTTCAGGTTG;

*Osteoprotegerin* (OPG): CGCTCGTGTTTCTGGACAT, CGGTCTTCCACTTTGCTGTA;

*Monocyte chemoattractant protein (MCP)-1*: CAGCCAGATGCAATCAATGCC, TGGAATCCTGAACCCACTTCT;

*Macrophage inflammatory protein (MIP)-1β*: AAGCTCTGCGTGACTGTCCT, GCTTGCTTCTTTTGGTTTGG; and

*GAPDH*: CTGGGCTACACTGAGCACC, AAGTGGTCGTTGAGGGCAATG.

### Western blot analysis

Cell lysates were subjected to sodium dodecyl sulfate-polyacrylamide gel electrophoresis, transferred to a nitrocellulose membrane, and immunoblotted with antibodies against Atg3, Atg5, Beclin-1, LC3-I/II, PARP, caspase-3, caspase-8, caspase-9, and phosphorylated (p) or non-phosphorylated Stat3 and Jak1. β-actin levels served as a loading control.

### Mouse models, flow cytometry analysis, radiography, and immunohistochemical staining

Six to eight week-old female CB.17 SCID mice obtained from Charles River Laboratories were housed and maintained in American Association of Laboratory Animal Care-accredited facilities. The studies were approved by the Institutional Animal Care and Use Committee of MD Anderson. 5 × 10^5^ MM cells alone or in combination with 5 × 10^5^ green fluorescent protein (GFP)-labeled mature adipocytes were directly injected into the mouse femur [49]. Melphalan treatment (50 μg/mouse) was given after 2 weeks. For MM xenograft mouse models, mice were injected subcutaneously with 1 × 10^6^ ARP-1 cells. 4 weeks later when palpable tumors (5 mm in diameter) developed, melphalan treatment was given every 3 days for 3 weeks. Control mice received equal amounts of PBS. Tumors were measured twice a week with calipers and tumor volumes (mm^3^) were calculated as (width^2^ × length)/2. Mice were euthanized when moribund or when subcutaneous tumors reached 15 mm in diameter.

BM cells from mouse femurs were flushed out, and the human CD138^+^ or CD138^−^ subset was isolated using anti-human CD138-coated magnetic beads. Cells were then labeled with the antibodies, and analyzed with a BD LSRFortessa flow cytometer. Mouse sera were collected for ELISA analysis. To measure size of lytic bone lesions, radiographs were scanned with a Faxitron X-ray cabinet (IL). For histologic analyses, mice femurs were fixed in 10% neutral-buffered formalin for 18 hours. Formalin-fixed, paraffin-embedded sections of bones were deparaffinized [15]. Slides were stained with anti-perilipin or anti-CD138 antibodies, and counterstained with hematoxylin.

### Statistical analysis

Experimental values were expressed as means ± standard errors of the mean unless indicated otherwise. Statistical significance was analyzed with SPSS version 10.0 software using unpaired Student's *t*-tests and a one-way analysis of variance. A P value ≤ 0.05 was considered statistically significant. All results were reproduced in at least three independent experiments.
